# *Lactobacillus rhamnosus* GG cell-free supernatant as a novel anti-cancer adjuvant

**DOI:** 10.1186/s12967-023-04036-3

**Published:** 2023-03-14

**Authors:** Rossella Salemi, Silvia Vivarelli, Daria Ricci, Marina Scillato, Maria Santagati, Giuseppe Gattuso, Luca Falzone, Massimo Libra

**Affiliations:** 1 Department of Biomedical and Biotechnological Sciences, Section of General Pathology, Clinics and Oncology, University of Catania, Catania, Italy; 2 Present Address: Department of Biomedical and Dental Sciences, Morphological and Functional Imaging, Section of Occupational Medicine, University of Messina, Messina, Italy; 3 Department of Biomedical and Biotechnological Sciences, Section of Microbiology, University of Catania, Catania, Italy; 4 Epidemiology and Biostatistics Unit, Istituto Nazionale Tumori IRCCS Fondazione G. Pascale, Naples, Italy; 5 Research Center for Prevention, Diagnosis, and Treatment of Cancer, University of Catania, Catania, Italy

**Keywords:** *Lactobacillus rhamnosus GG*, LGG, Cancer, Adjuvant, Combination therapies

## Abstract

**Background:**

Gut microbiota modulation has been demonstrated to be effective in protecting patients against detrimental effects of anti-cancer therapies, as well as to improve the efficacy of certain anti-cancer treatments. Among the most characterized probiotics, *Lactobacillus* *rhamnosus GG* (LGG) is currently utilized in clinics to alleviate diarrhea, mucositis or intestinal damage which might be associated with several triggers, including *Clostridium difficile* infections, inflammatory gut diseases, antibiotic consumption, chemotherapy or radiation therapy. Here, we investigate whether LGG cell-free supernatant (LGG-SN) might exert anti-proliferative activity toward colon cancer and metastatic melanoma cells. Moreover, we assess the potential adjuvant effect of LGG-SN in combination with anti-cancer drugs.

**Methods:**

LGG-SN alone or in combination with either 5-Fuorouracil and Irinotecan was used to treat human colon and human melanoma cancer cell lines. Dimethylimidazol-diphenyl tetrazolium bromide assay was employed to detect cellular viability. Trypan blue staining, anti-cleaved caspase-3 and anti-total versus anti-cleaved PARP western blots, and annexin V/propidium iodide flow cytometry analyses were used to assess cell death. Flow cytometry measurement of cellular DNA content (with propidium iodide staining) together with qPCR analysis of cyclins expression were used to assess cell cycle.

**Results:**

We demonstrate that LGG-SN is able to selectively reduce the viability of cancer cells in a concentration-dependent way. While LGG-SN does not exert any anti-proliferative activity on control fibroblasts. In cancer cells, the reduction in viability is not associated with apoptosis induction, but with a mitotic arrest in the G2/M phase of cell cycle. Additionally, LGG-SN sensitizes cancer cells to both 5-Fluorouracil and Irinotecan, thereby showing a positive synergistic action.

**Conclusion:**

Overall, our results suggest that LGG-SN may contain one or more bioactive molecules with anti-cancer activity which sensitize cancer cells to chemotherapeutic drugs. Thus, LGG could be proposed as an ideal candidate for ground-breaking integrated approaches to be employed in oncology, to reduce chemotherapy-related side effects and overcome resistance or relapse issues, thus ameliorating the therapeutic response in cancer patients.

**Supplementary Information:**

The online version contains supplementary material available at 10.1186/s12967-023-04036-3.

## Background

Gut microbiota (GM) is composed of a plethora of different microorganisms (more than 100,000 trillion). Among them, bacteria are the most extensively studied [1]. Growing number of studies demonstrated that intestinal microbiota composition deeply affects human health [2, 3]. Regarding cancer, while certain detrimental bacterial species may induce cellular transformation triggering both local and systemic inflammation, other ones protect the host against tumor development, for example by improving the immune system functionality [4, 5]. Late diagnosis, resistance to therapy, recurrence and relapse are appalling issues which need to be overcome with the identification of new effective targeting strategies, especially for colon cancer, melanoma or lung cancer [6–9]. Pivotally, several approaches to modulate GM composition are currently used in clinics to ameliorate both adherence to treatments and anti-cancer therapy outcomes [10–14].

*Lactobacillus rhamnosus GG* (LGG), originally isolated from healthy human fecal samples in 1985 (by Gorbach and Goldwin, GG), and whose genome was fully sequenced in 2009, represents the most widely tested probiotic strain, both preclinically and clinically [15, 16]. As comprehensively outlined in a recent review by Lucio Capurso [17], in over 30 years of research, LGG demonstrated to be a very robust strain, able to survive to acidic gastric pH, to firmly adhere to the intestinal mucosa, as well as to produce epithelial-protective biofilms. Additionally, LGG efficiently shields non-transformed intestinal cells from varied sources of stressors (mechanicals and chemicals). Also, LGG efficiently contrasts intestinal microbial pathogen proliferation. Importantly, LGG may induce T helper 1 host immune response, thereby reducing gut inflammatory diseases and increasing anti-tumor targeting immunity [17]. Given all these proven properties, LGG is effectively employed in clinics to contrast diarrhea, leaky gut and/or mucositis which may be associated with: *Clostridium difficile* infections, inflammatory gut diseases, antibiotics, or anti-cancer therapies, but also non-alcoholic steatohepatitis, cystic fibrosis and many other conditions [18].

LGG is safely administered to children, elderly and immunocompromised individuals [19–21]. For this reason, it represents an ideal probiotic to be proposed as adjuvant in oncology [18]. The peculiarity of LGG is that several preclinical studies demonstrated its capability to effectively arrest cancer cell growth, both in vitro and in vivo (in several cancer types including oral, colon, cervical, breast) [4]. Despite all the existing observations, it is unclear yet whether LGG-derived anti-cancer active biomolecule(s) might be located within the bacterial cell, or attached to the surrounding wall (including pili), or actively secreted [4].

This work aims to characterize the effect of cell-free LGG supernatant (LGG-SN) on the growth of cancer cells versus non-transformed ones. Three colon cancer cell lines (HCT-116, Caco-2, HT-29) and one melanoma cell line (A375) have been used in this study in order to evaluate whether LGG-SN might affect both local-colon and intestine-distant tumors. Importantly, in light of future translational application, the effect of LGG-SN in combination with anti-cancer drugs has also been evaluated. Overall, the results from this study will pose valuable bases to further clarify whether LGG use might be a suitable integrated approach in oncology and what is the nature of the secreted LGG-component(s) able to selectively induce the observed anti-cancer activity.

## Methods

### Cell lines and culture

HCT-116, Caco-2, HT-29 human colon cancer cell lines, and A375 malignant melanoma cells were purchased from American Type Culture Collection (Manassas, VA, USA). Non-cancer human primary cutaneous fibroblasts were kindly provided by Professor Salvatore Travali, University of Catania (Italy). HCT-116, HT-29 and A375 cells were grown in Roswell Park Memorial Institute Medium (RPMI-1640; Sigma-Aldrich, St. Louis, MO, USA), while Caco-2 and Fibroblasts were cultured in Minimum Essential Medium (MEM; Sigma-Aldrich, St. Louis, MO, USA). All culture media were supplemented with 2 mmol/l L-glutamine (L-Glut), 100 IU penicillin, 100 μg/ml streptomycin and 10% heat-inactivated Fetal Bovine Serum (FBS, Sigma-Aldrich, St. Louis, MO, USA). Cells were maintained in a humidified, 37 °C and 5% CO_2_ incubator and used within 15 passages after thawing.

### LGG growth condition and cell-free supernatant production

Probiotic strain *Lactobacillus rhamnosus GG* (ATCC 53,103, LGG) was provided by Dicofarm Spa (Rome, Italy). LGG cell-free supernatant (LGG-SN) from live LGG culture was obtained through several steps summarized in Table 1.

**Table 1:** Procedure for the generation of LGG conditioned media

	LGG biomass	LGG conditioned medium	LGG-free conditioned medium complete (LGG-SN)	Lyophilized LGG supernatant (Lyoph LGG-SN)
Medium	MRS	RPMI-1640	RPMI-1640	Resuspended in sterile ddH_2_O
Supplements	n.a	n.a	FBS (10% v/v); L-Glut (2 mM)	Stock concentration 5% (v/v) in complete RPMI-1640 (with 10% FBS and 2 mM L-Glut)
Growth conditions	ON 37 °C	5 h 37 °C	n.a	n.a
Incubation	Exponential bacteria growth	Living bacteria biomass	Bacteria free; Filter-sterilized	Bacteria free
pH range	5.0–5.5	6.5–7.0	6.5–7.5	7.5–8.0
OD 600 nm	1.88 ± 0.2	1.91 ± 0.3	n.a	n.a
Bacteria count (CFU/ml)	2.9 × 10^9^	2.4 × 10^10^	n.a	n.a

n.a., not associated; MRS, de Man, Rogosa and Sharpe; RPMI-1640, Roswell Park Memorial Institute Medium

LGG was grown in de Man, Rogosa and Sharpe (MRS) agar (Oxoid, Thermo Fisher Scientific Inc., Waltham, MA, USA), incubated for 48 h at 37 °C under anaerobic conditions, using the GasPakEZ Gas Generating Pouch Systems (BD Diagnostics, Franklin Lakes, NJ, USA). LGG cultures grown anaerobically for 48 h at 37 °C in MRS broth (Oxoid) were harvested by centrifugation (5,000 × g for 15 min, at 4 °C), and the cells were washed twice with a sterile solution of 0.85% NaCl (w/v) (Sigma-Aldrich, St. Louis, MO, USA). Subsequently, 200 µl of 10^8^ CFU/ml LGG bacteria starter culture were inoculated in 500 ml of MRS broth and further incubated at 37 °C for 20 h without agitation. Once reached late-exponential growth phase, at 2.9 × 10^9^ CFU/ml, live LGG was separated from MRS broth through a centrifugation step at 7,000 RPM for 30 min, at 4 °C. Hence, LGG bacterial pellet was washed twice with sterile Phosphate Buffered Saline (PBS) solution (to eliminate MRS medium residuals) and resuspended in 500 ml of sterile RPMI-1640. LGG bacterial suspension was incubated in RPMI-1640 for 5 h at 37 °C without agitation, to obtain LGG conditioned medium. The LGG-conditioned suspension was subsequently centrifuged at 7,000 RPM for 30 min at 4 °C, conditioned medium was separated from the bacterial pellet, and filtered-sterilized with a bottle filtration unit, 0.22 µm (Merck Millipore, Darmstadt, Germany). LGG conditioned cell-free RPMI-1640 supernatant was supplemented with FBS (final concentration 10% v/v) and L-Glut (final concentration 2 mM) to obtain the complete LGG conditioned supernatant (cell-free and sterile), or LGG-SN, used in the in vitro experiments (and considered 90% v/v concentrated, stock concentration). Control MRS LGG cell-free supernatant was obtained by incubating bacteria (corresponding to 2.9 × 10^9^ CFU/ml) in MRS for 5 h at 37 °C without agitation. LGG suspension was centrifuged at 7,000 RPM for 30 min at 4 °C, MRS-conditioned supernatant was separated from the bacterial pellet, and filtered-sterilized with a bottle filtration unit, 0.22 µm. The obtained sterile LGG conditioned cell-free MRS supernatant was supplemented with FBS (final concentration 10% v/v) and L-Glut (final concentration 2 mM) to obtain the complete LGG conditioned MRS (sterile, cell-free) control medium (90% v/v, stock concentration).

LGG lyophilized cell-free and sterile supernatant (lyoph-LGG-SN) was obtained resuspending one bottle of LGG (cell-free) lyophilized broth (lot 2S20X-WBR085-19, Dicofarm Spa Rome, Italy) in 1 ml of sterile distilled water (ddH_2_O). The solution (and derived dilutions in ddH_2_O) was then diluted to 5% (v/v) in sterile complete RPMI-1640 (with 10% FBS and 2 mM L-Glut), to obtain complete lyoph-LGG-SN stock solution used in the in vitro experiments.

### Cell viability assays

The 3-(4,5-Dimethylthiazol-2-yl)-2,5-diphenyl tetrazolium bromide (475,989, MTT, Sigma-Aldrich, St. Louis, MO, USA) assay was used to assess cellular viability. For treatments with LGG-SN, cells were seeded in triplicate samples into a 96-well plate (3,000 cells per well for HT-29, HCT-116, A375, or 5,000 cells per well for Caco-2 and fibroblasts), treated with LGG-SN added to complete RPMI-1640 from 90 to 10% (v/v), control cells were treated with the same percentage of complete RPMI-1640 without LGG-SN (control medium, CTRL). For treatments with Lyoph-LGG-SN, tumor cells were treated with Lyoph-LGG-SN in a range between 5% and 0.001% (v/v) in complete RPMI-1640, control cells were treated with the same maximum percentage of diluent (5% ddH_2_O, v/v) in complete medium. For anti-cancer treatments, cells were treated in combination with different concentrations of LGG-SN (0%, 50% and a higher concentration corresponding to specific IC_50_ of the tested cell line) and 5-Fluorouracil (5-FU; F6627, Sigma-Aldrich, St. Louis, MO, USA), with concentrations ranging from 5.0 × 10^–4^ to 7.6 × 10^–9^ M or Irinotecan (IRN; I1406, Sigma-Aldrich, St. Louis, MO, USA), with concentrations ranging from 2.0 × 10^–4^ to 3.1 × 10^–9^ M, as well as in combination with different concentrations of Lyoph-LGG-SN (0, 0.3%, 1% and 3%) and 5-FU with concentrations ranging from 5.0 × 10^–4^ to 5.0 × 10^–7^ M.

Following 48 h of incubation, cells were assessed for their viability by adding 0.5 µg/ml MTT per well. Insoluble formazan crystals were dissolved by adding an acid-isopropanol stop solution (0.04N HCl). Absorbance was measured at 610 nm, using the Tecan-Sunrise microplate reader (Tecan, Männedorf, Switzerland). To assess synergy between anti-cancer treatments and either LGG-SN or Lyoph-LGG-SN, viability results were analyzed through the free online tool Synergy Finder 2.0 [22]. For each combination analyzed, data outcomes were presented as inhibition percentage matrices and synergy score matrices. In particular, the Highest Single Agent (HSA) model was applied to score the synergy, which states that the expected combination effect is equal to the higher effect obtained with individual treatments. Therefore, any additional effect over the higher single compound treatment has been considered as HSA synergy [23]. For trypan blue count, from 1.0 × 10^5^ cells per well (for HT-29, HCT-116 and A375) to 2.0 × 10^5^ cells per well (for Caco-2 and fibroblasts) were seeded in 12 well plates and treated with different concentrations of LGG-SN. After 48 h of incubation and harvesting, cellular samples were mixed 1:1 with 0.4% Trypan Blue (15250061, Thermo Fisher Scientific, Waltham, MA, USA). Cells permeable to Trypan Blue were counted as dead. Counts were performed by using a Bürker chamber and the Eclipse Ts2 inverted microscope (Nikon, Melville, NY, USA). Doubling times for CTRL and LGG-SN 90% treated samples have been calculated with the following formula:

$$Doubling\, time (hours)=\frac{48 hours\times ln(2)}{ln(\frac{{N}_{f}}{{N}_{i}})}$$

where 48 h indicates the cell culture duration, N_f_ indicates the final number of viable cells at the endpoint and N_i_ indicates the initial seeding density.

### Total RNA extraction, cDNA synthesis and quantitative RT-PCR analyses

For total RNA extraction, up to 3 × 10^6^ cancer cells were harvested and total RNA was isolated using GeneJET RNA Purification Kit (K0731, Thermo Fisher Scientific, Waltham, MA, USA). For cDNA synthesis, 3 µg of the total RNA was reverse-transcribed with Super-Script IV Reverse Transcriptase (18090010, Thermo Fisher Scientific, Waltham, MA, USA).

The template cDNA was amplified using the primer pairs reported in Table 2.

**Table 2:** Primer pairs for RT-PCR experiments

Gene ID	Primer F	Primer R	Product Size (bp)
*Cyclin A (CCNA2)*	CCAGTCCACGAGGATAGCTC	GCCTGCGTTCACCATTCATG	364
*Cyclin B (CCNB1)*	AAGAGCTTTAAACTTTGGTCTGGG	CTTTGTAAGTCCTTGATTTACCATG	319
*Cyclin D (CCND1)*	CCGAGAAGCTGTGCATCTAC	GGCGGTAGTAGGACAGGAAG	324
*GAPDH*	AGAAGGCTGGGGCTCATTTG	AGGGGCCATCCACAGTCTTC	258

Luminaris Color HiGreen qPCR Master Mix, high ROX was used for quantitative RT-qPCR (q-RT-PCR; K0361, Thermo Fisher Scientific, Waltham, MA, USA). 7300 Real-Time PCR System was employed to detect cDNA amplification (Thermo Fisher Scientific, Waltham, MA, USA). Expression levels of target genes were normalized to the mean expression levels of human *GAPDH* housekeeping gene. In particular, the 2^(−ddCt)^ relative quantification method was employed [24].

### Protein lysates preparation, quantification and immunoblot analyses

For protein extraction, up to 5 × 10^6^ cells were harvested. The collected cells were lysed using nonidet-P40 buffer (FNN0021, Thermo Fisher Scientific, Waltham, MA, USA) supplemented with protease and phosphatase inhibitors (11836170001 and 4906845001, Roche Diagnostics, Indianapolis, IN, USA). Protein concentration was determined with Bradford assay (5000201, Bio-Rad Laboratories, Hercules, CA, USA). Protein samples were separated using Mini-Protean precast gels and gel-electrophoresis system, and protein gels were transferred using Trans-Blot Turbo transfer system (4561085 and 1704158, both from Bio-Rad Laboratories, Hercules, CA, USA). Nitrocellulose membranes were blocked with 5% of non-fat dry milk diluted in TBS-T buffer (0.1% Tween 20, 20 mM Tris–HCl pH 7.6, 137 mM NaCl). Immunoblotting analyses were performed using the following antibodies, according to manufacturer’s instructions: anti-cleaved-Caspase-3 (1:1,000 dilution; Rabbit, Cell Signaling Technology, Danvers, MA, USA, CST-9664); anti-total/cleaved-PARP-1 (1:1,000 dilution; Rabbit, Cell Signaling Technology, Danvers, MA, USA, CST-9532); anti-β-Tubulin (1:5,000 dilution; loading control, Rabbit, Abcam, Cambridge, United Kingdom, ab6046); anti-β-Actin (1:10,000 dilution, Mouse, Sigma-Aldrich, St. Louis, MO, USA, a1978); Goat Anti-Rabbit IgG Antibody, Fc, HRP conjugate (1:3,000 dilution; Chemicon International, Fisher Scientific, Waltham, MA, USA, AP156P). Enhanced chemiluminescence signals (1705060, ECL kit, Bio-Rad Laboratories, Hercules, CA, USA) were acquired with the ChemiDoc Touch Imaging System (Bio-Rad Laboratories, Hercules, CA, USA).

### Flow cytometry analyses

6 × 10^5^ cancer cells were seeded in 10 cm culture dishes and treated either with LGG-SN 90% or with CTRL medium for 48 h. 5 × 10^–7^ M Vincristine (VIN, V8388, Sigma-Aldrich, St. Louis, MO, USA) treatment in complete RPMI-1640 medium was used as positive control for cell cycle analysis. In fact, VIN disrupts mitotic spindle formation thereby blocking cells in G2/M phase [25]. Moreover, 0.5 µg/ml puromycin (PURO, P4512, Sigma-Aldrich, St. Louis, MO, USA) treatment in complete RPMI-1640 medium was used as cell death control. Following treatments, cells were harvested through cell scraping, washed twice in PBS and kept on ice. For cell cycle analysis, upon centrifugation at 1,200 rpm for 5 min at room temperature, and PBS removal, the cell pellet was resuspended in ice-cold 70% ethanol solution. After an incubation overnight at 4 °C, cells were pelleted through centrifugation at 1,200 rpm for 5 min at 4 °C, washed in PBS to remove all the ethanol, and cell pellets were subsequently resuspended in Propidium Iodide (PI) staining solution containing PBS with 0.1% (v/v) Triton X-100, 10 μg/ml PI (P4170, Sigma-Aldrich, St. Louis, MO, USA), 100 μg/ml DNase-free RNase A (R5125, Sigma-Aldrich, St. Louis, MO, USA). Cells in PI solution were incubated 10 min at 37 °C in the dark. Instead, for cell death analysis, harvested cells were immediately incubated with Alexa Fluor 488 Annexin V/Dead Cell Apoptosis Kit components (V13241, Thermo Fisher Scientific, Waltham, MA, USA), following manufacturer’s instruction.

Subsequently, samples were analyzed with Amnis Flow Sight Imaging Flow Cytometer (Luminex, USA). Fluorescence intensity of PI and/or Annexin V was measured by using 488 nm laser. Flow cytometric gating was used to select focused single cells and the mean fluorescence intensity of treated cells was compared with that of CTRL treated cells. Amnis IDEAS software version 6.1 (Luminex, Austin, TX, USA) was used for data analyses. Amnis cell cycle wizard was used to quantify PI fluorescence detected in Channel 5 (640–745 nm) in correlation with the DNA cellular content and, hence with the corresponding cell cycle phase. Whereas, for cell death analysis single cells in focus were gated based on fluorescence intensity detected in Channel 2 (480–560 nm, Annexin V signal) in function of fluorescence intensity detected in Channel 5 (640–745 nm, PI signal). Early apoptotic cells are Annexin V positive, necrotic cells are PI positive, advanced apoptotic cells are both Annexin V and PI positive, while live cells show no fluorescence.

### Statistical analyses

Statistical analyses were performed using GraphPad Prism version 9.0 for Windows (GraphPad Software, La Jolla, CA, USA). Results were presented as Mean ± standard deviation (SD). Single parameter comparisons between two groups were conducted using two-tailed unpaired Student’s t-test (parametric data) or Mann–Whitney’s U-test (non-parametric data). Single parameter comparisons between three or more groups were performed using one-way analysis of variance (ANOVA) with Tukey’s or Dunnett’s multiple comparison tests (parametric data) or Kruskal–Wallis H-test (non-parametric data). Multiple parameter comparisons between two groups were performed using two-way ANOVA with Tukey’s multiple comparison test. Differences were considered significant with p < 0.05; being: *p < 0.05; **p < 0.01; ***p < 0.001; ****p < 0.0001.

## Results

### LGG cell free supernatant (LGG-SN) selectively reduces the viability of cancer cells in a concentration-dependent manner.

To assess the effect of LGG-SN on cellular growth, five different cell lines have been employed. Three cell lines are colon adenocarcinomas (HT-29, HCT-116 and Caco-2), whereas A375 cells derive from a cutaneous malignant melanoma. Additionally, non-tumor fibroblast cells have been also tested as non-transformed control. In this concentration–response experiment, 9 concentrations of LGG-SN have been tested, from 90 to 10% v/v, (delta difference between doses is 10% v/v), plus the CTRL (0% v/v) which is complete growth medium. The MTT readout was performed 48 h after treatment.

**Fig. 1:**
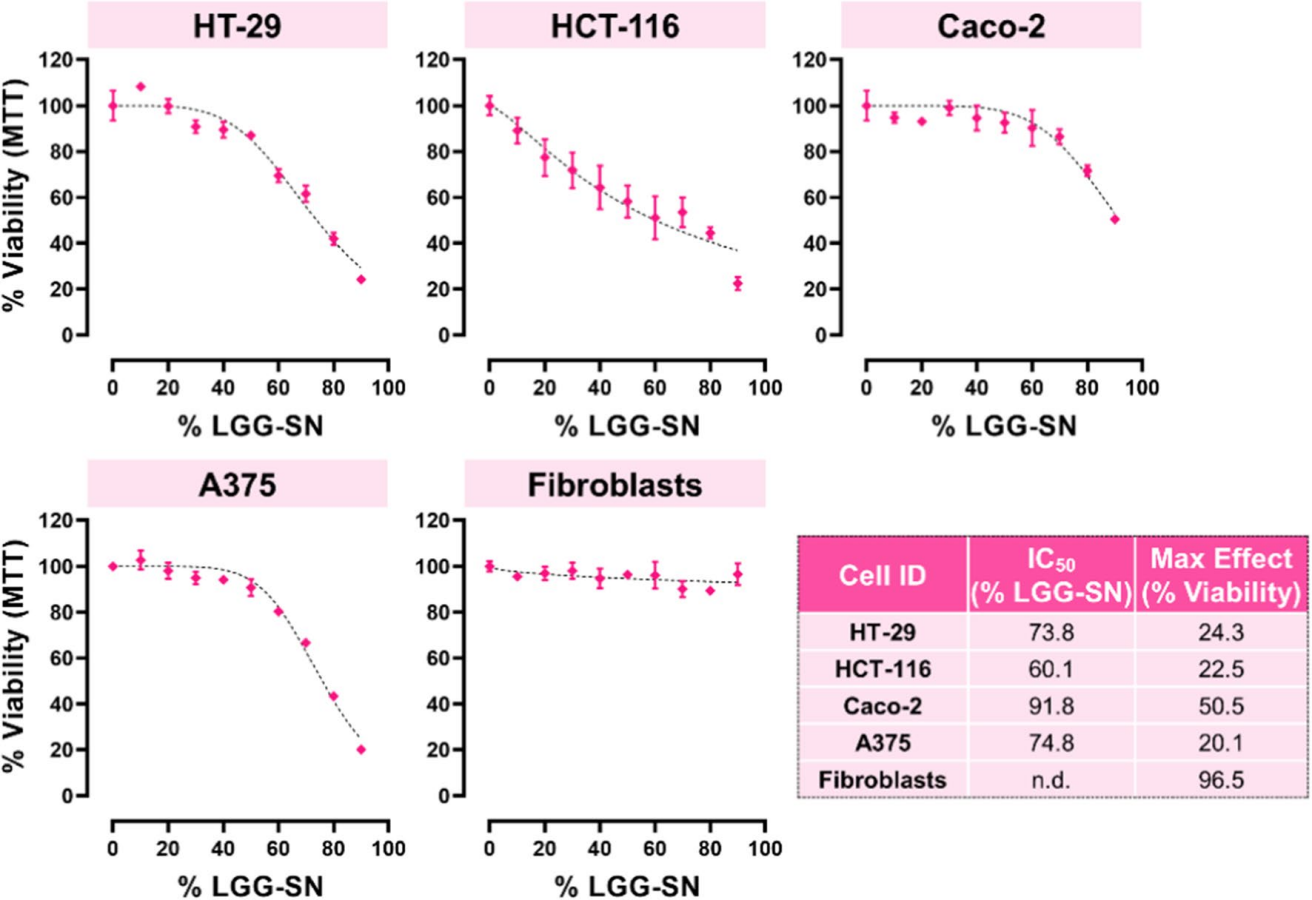
LGG cell free supernatant (LGG-SN) selectively reduces the viability of cancer cells in a concentration-dependent manner. Concentration–response plots for HT-29, HCT-116, Caco-2, A375 and Fibroblasts treated with increasing concentrations of LGG-SN (up to 90% v/v). MTT assay readout reveals a concentration dependent decrease of cellular viability in the four cancer cell lines, but not in Fibroblasts. Table reported in the bottom right summarizes the IC_50_ values (% of LGG-SN, v/v) and maximum effect (% viability) calculated per each cell type tested. N = 3. Values are presented as Mean ± SD

As shown in Fig. 1, the maximum effect obtained in terms of residual viability at the maximum concentration of LGG-SN is strictly cell line dependent. Among tumor cells, Caco-2 are the less sensitive, with a residual viability of 50.5% with 90% (v/v) LGG-SN treatment. While, the other three cancer cell lines tested, including melanoma A375 cells, show a residual viability of about 20%. Interestingly, non-transformed fibroblasts are insensitive to LGG-SN treatment, and, even at the highest LGG-SN concentration tested (90% v/v), the measured viability in fibroblasts is still 96.5%. This result suggests that LGG-SN selectively reduces the viability of cancer cells, in a concentration dependent manner. Correspondingly, the relative IC_50_ values calculated for cancer cell lines range between 91.8% LGG-SN (v/v) for Caco-2 less-sensitive cells, to 73.8% LGG-SN (v/v) and 74.8 LGG-SN (v/v) for HT-29 and A375, respectively; and, finally 60.1% LGG-SN (v/v) for HCT-116 (Fig. 1).

### Cells treated with LGG-SN show a decrease in cell number not associated with concurrent cell death

**Fig. 2:**
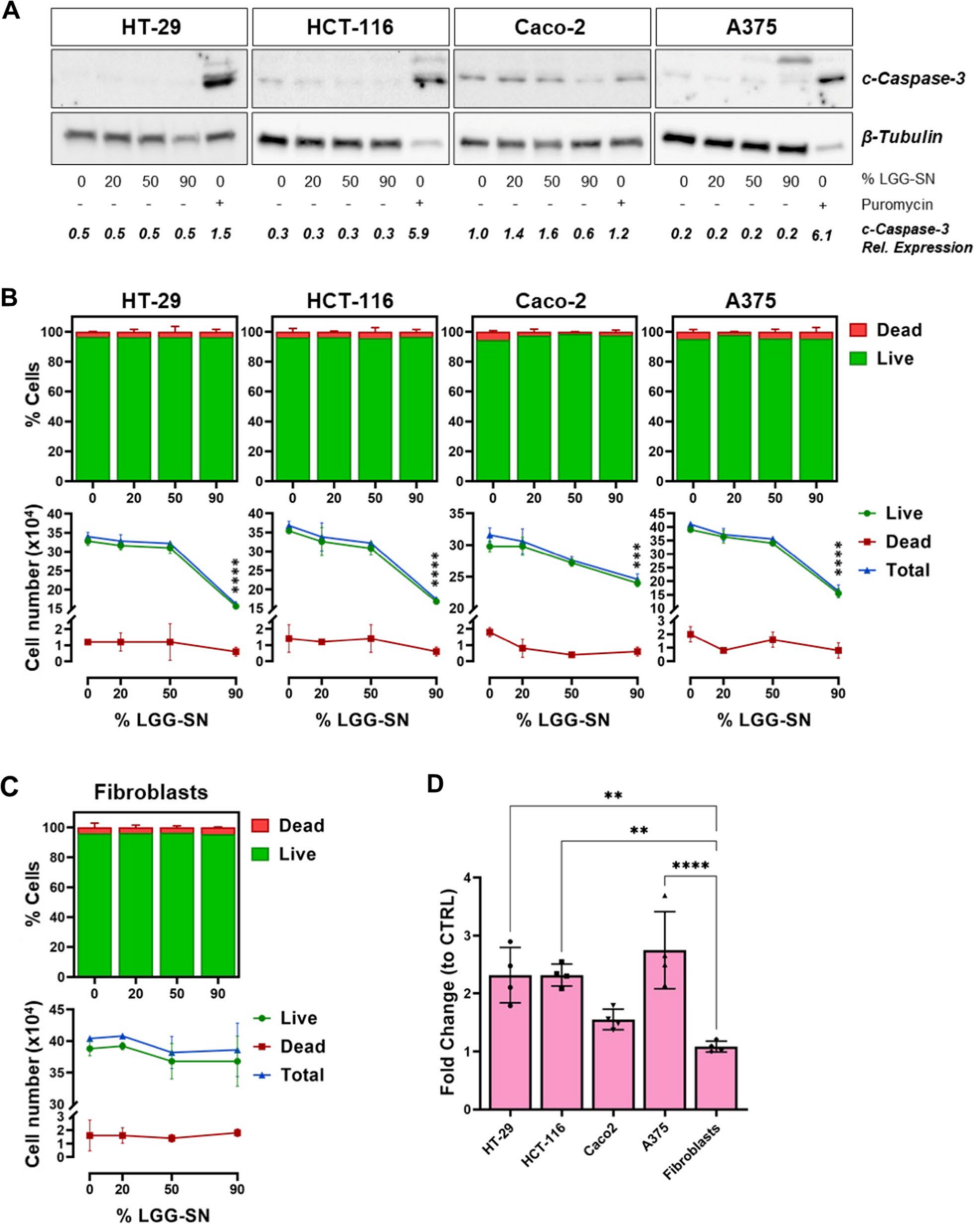
Cancer cells treated with LGG-SN show a decrease in cell number not associated with concurrent cell death. **A**. Immunoblot and densitometry of HT-29, HCT-116, Caco-2, A375 treated with 0% (CTRL), 20%, 50% and 90% v/v LGG-SN, or 0.5 µg/ml Puromycin (positive control treatment). Signal detected for cleaved Caspase 3 (c-Casp-3; 17–19 KDa) and β-Tubulin (normalization control, 48 KDa). **B**. Trypan blue count for HT-29, HCT-116, Caco-2, A375 treated with 0% (CTRL), 20%, 50% and 90% v/v LGG-SN. Top bar plots: percentage of live (green) and dead (red) cells per each concentration. Bottom plots: cell count (viable green, dead red, total blue) per each concentration of LGG-SN (0% v/v CTRL, 20%, 50% and 90% v/v). **C**. Trypan blue count for Fibroblasts treated with different concentrations LGG-SN. Top bar plots: percentage of live (green) and dead (red) cells per each concentration. Bottom plots: cell count (viable green, dead red, total blue) per each concentration of LGG-SN. **D**. Fold change of doubling time of 90% v/v LGG-SN over CTRL 0% v/v treatment. N = 3. Values are presented as Mean ± SD. Statistical significance was analyzed using ordinary one-way ANOVA and two-way ANOVA with Tukey's multiple comparisons test. **p < 0.01; ***p < 0.001; ****p < 0.0001; no asterisk = not significant

To assess whether the decrease in cell viability following LGG-SN treatment was associated with a concurrent increase in cell death, total protein samples from cancer cells treated for 48 h either with increasing concentration of LGG-SN or CTRL complete medium, or puromycin as positive control, were tested for the expression of cleaved Caspase-3 (c-Caspase-3), an effector of apoptosis. As shown in Fig. 2A, all four cancer cells did not show any increase in the production of cleaved Caspase 3 following LGG-SN treatment, even at the highest concentration, and in contrast with puromycin-treated controls. Furthermore, protein expression levels of 116 KDa full-length PARP (t-PARP) and 89 KDa Caspase-cleaved PARP (c-PARP) fragment have been also measured. As showed in Additional file 1: Fig. S1, in all 90% LGG-SN-treated samples, t-PARP levels are comparable with ones detected in CTRL samples, whereas c-PARP is not detectable. Contrariwise, in puromycin-treated control, t-PARP signal is about ten-fold reduced, whereas c-PARP signal is ten-fold increased when compared with both CTRL- and 90% v/v LGG-SN-treated samples. Accordingly, t-PARP/c-PARP ratio is > 1 in both CTRL-treated and LGG-SN-treated cells, while it is < 1 in puromycin-treated control. Overall, both c-Caspase-3 and PARP detection, support the exclusion of the involvement of apoptosis following LGG-SN treatment in cancer cells.

In order to analyze whether early apoptosis might be triggered, the protein expression of Annexin V, early marker of apoptosis, has been measured. As showed in Additional file 1: Fig. S2, flow cytometry results confirmed that there is no significant increase of Annexin V and/or PI staining in LGG-SN-treated samples compared with CTRL ones in none of the cell lines tested. Therefore, not only advanced apoptosis, but also early apoptosis and necrosis are not associated with viability reduction observed in tumor cells following LGG-SN treatment.

Additionally, Trypan cellular count showed a significant reduction in the number of total and viable cells treated with LGG-SN 90% v/v compared with 0% v/v CTRL. As further supported by Trypan blue count, such reduction in viable cell number is not associated with a concurrent increase in the number of dead cells, further confirming that LGG-SN treatment is not associated with a cytotoxic effect in cancer cells (Fig. 2B). In line with the viability assay results, non-transformed fibroblasts did not show a decrease in total or viable cell number upon LGG-SN treatment, nor an increase of cells counted as dead (Fig. 2C). Comparing the doubling time of the tumor cells with fibroblasts following 90% of LGG-SN (v/v) treatment, it is possible to notice that while all cancer cells show a reduction in their doubling time, fibroblasts continue to grow with no change of this parameter (with fold change equal to unitary, Fig. 2D). Individual doubling times measured in each cell line upon CTRL and 90% LGG-SN treatment are reported in Additional file 1: Fig. S3.

### Cell cycle analyses reveal G2/M block following LGG-SN treatment in cancer cells

**Fig. 3:**
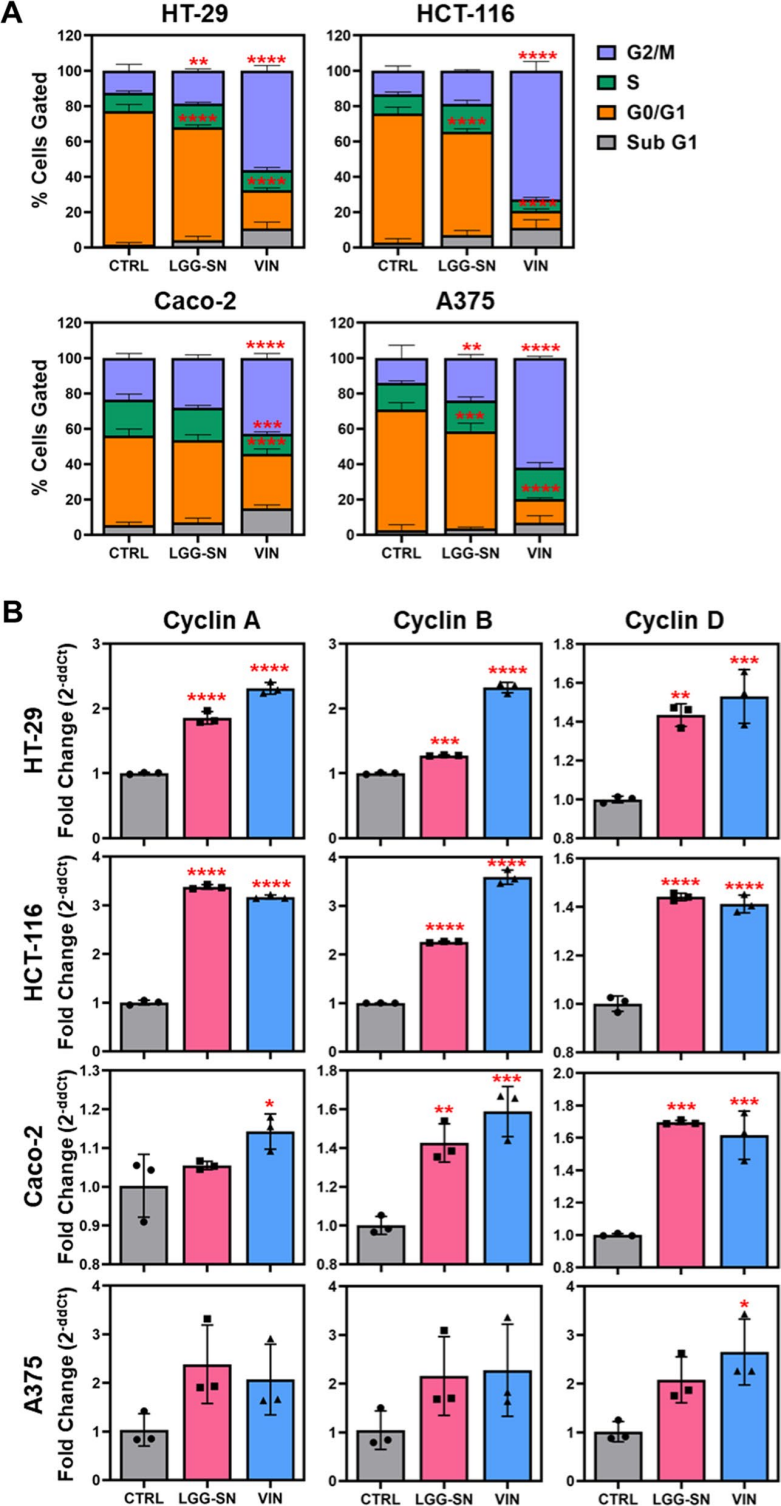
Cell cycle analyses reveal G2/M block upon LGG-SN treatment in cancer cells. HT-29, HCT-116, Caco-2, A375 treated with 0% v/v (CTRL), 90% v/v LGG-SN and 5 × 10^–7^ M Vincristine (VIN). **A**. Cell cycle bar plots: percentage of cells in G2/M (purple), S (green), G0/G1 (orange) and sub G1 (grey). **B**. q-RT-PCR analysis of Cyclin A (left), Cyclin B (middle), Cyclin D (right) expression in HT-29, HCT-116, Caco-2, and A375, GAPDH used as housekeeping, data are expressed as 2^−ddCt^ compared to CTRL. N = 3. Values are presented as Mean ± SD. Statistical significance was analyzed using ordinary one-way ANOVA with Dunnett's multiple comparisons test (q-RT-PCR plots) and two-way ANOVA with Tukey's multiple comparisons test (cell cycle plots). *p < 0.05; **p < 0.01; ***p < 0.001; ****p < 0.0001; no asterisk = not significant

To assess whether the reduction in cell viability observed in cancer cells with the MTT assay following LGG-SN treatment was associated with cell cycle modulation, the PI assay has been performed following 90% LGG-SN (v/v) treatment, in comparison with untreated CTRL, as well as VIN treatment used as positive control. Interestingly, following LGG-SN treatment for 48 h, HT-29, HCT-116 and A375 show a significant increase in the number of cells in G2/M phase and a concurrent decrease of cells in G0/G1 phase. Caco-2 cells show a trend of increase of cells in G2/M phase although not significant when compared with 0% LGG-SN (v/v) treated CTRL (Fig. 3A and Additional file 1: Fig. S4). Moreover, in agreement with cell death analyses, the percentage of cells in Sub-G1 phase, corresponding to the number of dead cells, was found unvaried following LGG-SN treatment. Additionally, the analysis of Cyclin A, Cyclin B and Cyclin D gene expression levels was also performed. As shown in Fig. 3B, all cells showed an increase in the expression of all three cyclin genes, significant in the most LGG-SN sensitive colon cancer cell lines (HT-29, HCT-116 and Caco-2). Overall, cell cycle analysis demonstrates that LGG-SN treatment is associated with a reduction in viability, as well as an arrest in G2/M phase of the cell cycle, thereby suggesting that LGG-SN exerts a cytostatic effect in cancer cells.

### LGG-SN in combination with 5-FU and IRN shows a synergistic effect in cancer cells

**Fig. 4:**
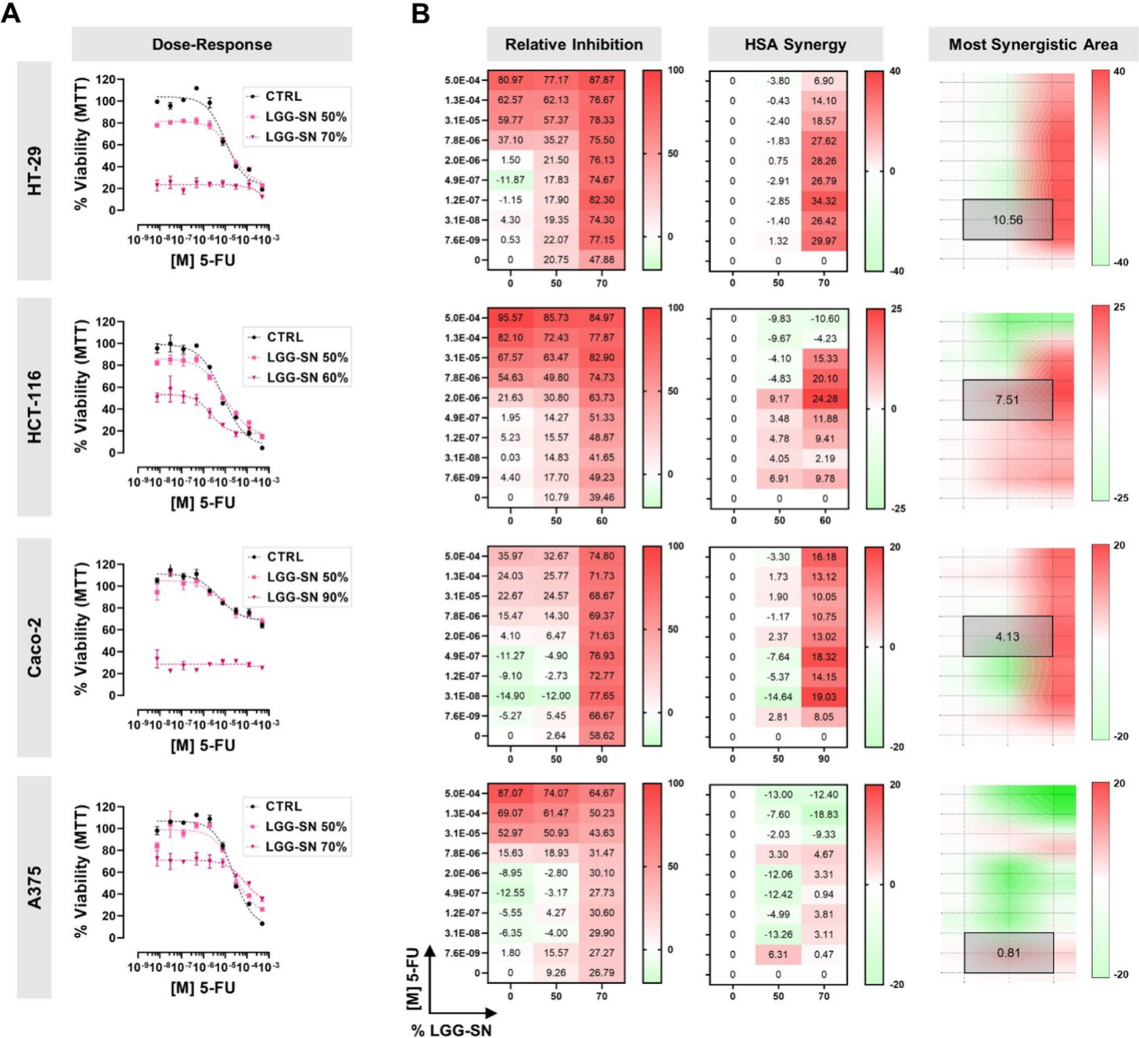
LGG-SN in combination with 5-Fluorouracil (5-FU) shows a synergistic effect in cancer cells. **A**. HT-29, HCT-116, Caco-2, A375 concentration–response plots. Cells treated with 5-FU (from 7.6 × 10^–9^ M to 5.0 × 10^–4^ M) in combination with different LGG-SN concentrations (50% and 70% v/v). **B**. HT-29, HCT-116, Caco-2, A375 relative inhibition matrices (left), Highest Single Agent (HSA) synergy matrices (middle), synergy surface with most synergistic area (black square, with corresponding HSA synergy score). N = 3. Values are presented as Mean ± SD

It was further analyzed whether LGG-SN in combination with cytotoxic drugs might show any additive effect. To this purpose, tumor cells were treated with two different concentrations of LGG-SN in combination with several concentrations of two diverse anti-cancer drugs currently used in clinics: 5-FU (5.0 × 10^–4^ to 7.6 × 10^–9^ M) and IRN (2.0 × 10^–4^ to 3.1 × 10^–9^ M). Figure 4 shows the results obtained for 5-FU. In particular, in Fig. 4A it is possible to observe that cancer cells treated with 5-FU in combination with either a concentration of LGG-SN 50% v/v (light purple curves) or around the IC_50_ calculated for each cell line (i.e., 90% for Caco-2, 70% for HT-29 and A375, 60% for HCT-116; dark purple curves), show a decrease in viability compared with the concentration–response curve obtained with 5-FU alone (black curves). Per each cancer cell line, Fig. 4B shows the calculated inhibition matrix, as well as the results expressed in terms of HSA synergy scores. For all three colon cancer cell lines tested it is possible to observe maximum synergy scores above the cutoff value of 10 (i.e., 34.32 for HT-29, 24.28 for HCT-116, 19.03 for Caco-2), meaning that the combined treatments have a synergistic effect compared with single drug treatments in colon cancer cells, while the effect is observed to lesser extent in A375 melanoma cells (maximum synergy of 6.31). Synergy distribution plots (Fig. 4B, left) identify where in the matrix is located the maximum synergistic area and the corresponding HSA synergy score (mean values of 10.56 for HT-29, 7.51 for HCT-116, 4.13 for Caco-2 and 0.81 for A375).

**Fig. 5:**
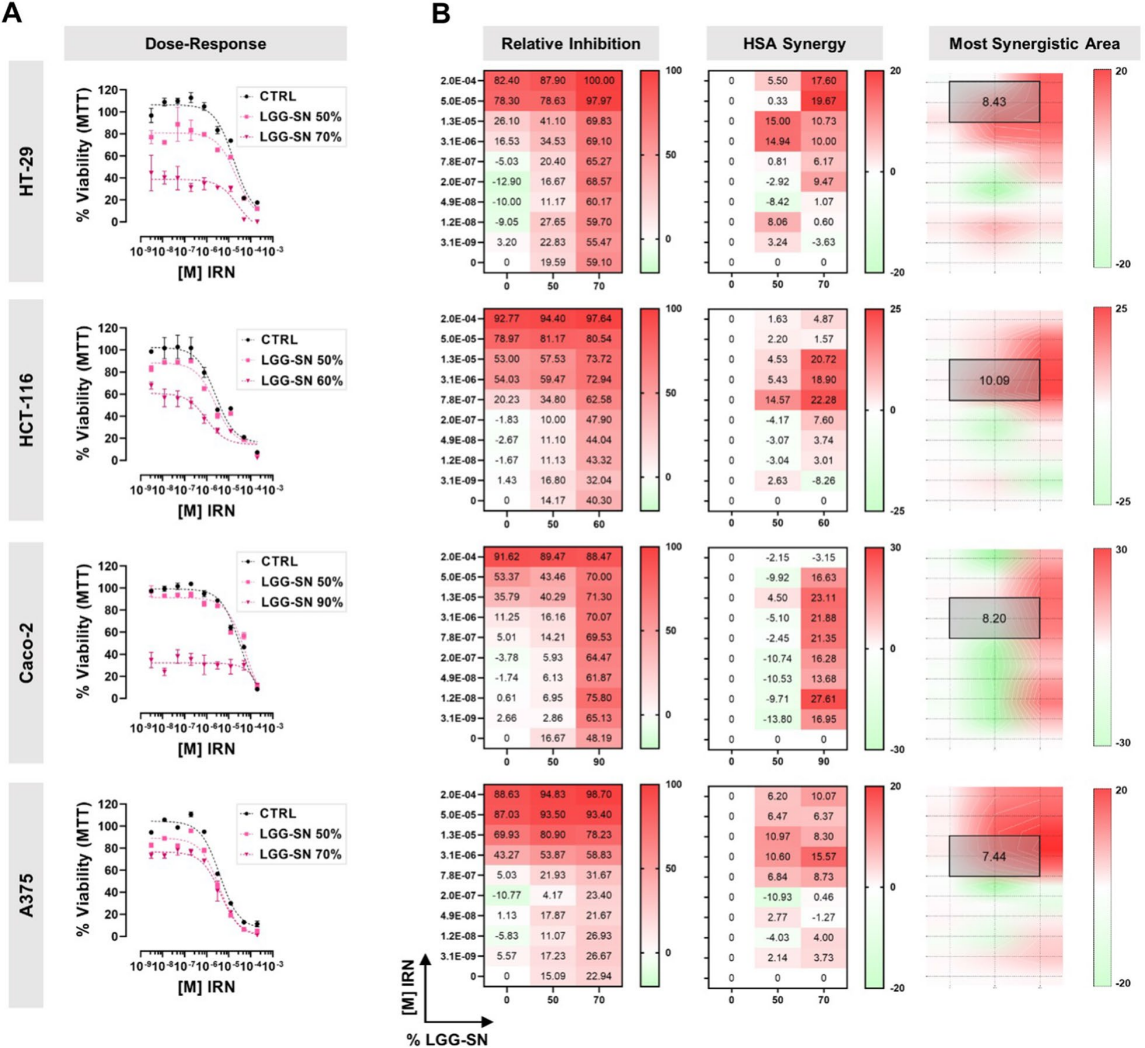
LGG-SN in combination with Irinotecan (IRN) shows a synergistic effect in cancer cells. **A**. HT-29, HCT-116, Caco-2, A375 concentration response plots. Cells treated with IRN (from 3.1 × 10^–9^ M to 2.0 × 10^–4^ M) in combination with different LGG-SN concentrations (50% and 70% v/v). **B**. HT-29, HCT-116, Caco-2, A375 relative inhibition matrices (left), Highest Single Agent (HSA) synergy matrices (middle), synergy surface with most synergistic area (black square, with corresponding HSA synergy score). N = 3. Values are presented as Mean ± SD

The same approach was followed to analyze the combined effect of LGG-SN with IRN, and results are shown in Fig. 5A. In particular, the maximum synergy scores were 19.67 in HT-29 cells, 22.28 in HCT-116, 27.41 in Caco-2 and 15.57 in A375. While, the most synergistic areas showed mean values of 8.43 for HT-29, 10.09 for HCT-116, 8.20 for Caco-2 and 7.44 for A375 (Fig. 5B). Overall, the results obtained demonstrated that LGG-SN shows synergistic effects in combination with both 5-FU and IRN anti-cancer drugs, in all the cancer cell lines tested. The different synergy scores calculated may be explained by cell-specific sensitivity to the compounds.

### Lyoph-LGG-SN selectively reduces the viability of cancer cells in a concentration-dependent manner, and shows a synergistic effect in combination with 5-FU

**Fig. 6:**
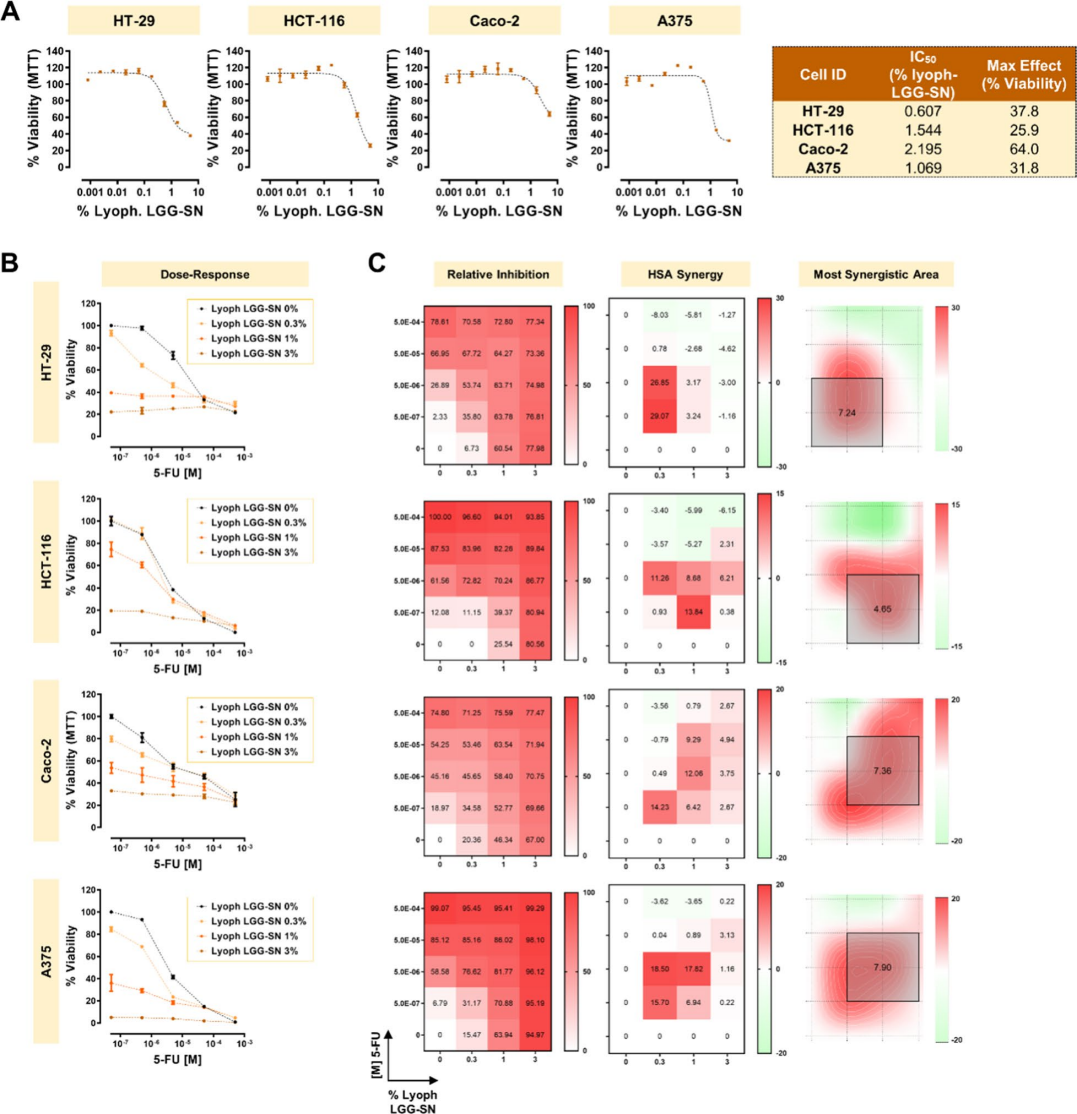
Lyophilized cell-free LGG-SN (Lyoph-LGG-SN) selectively reduces the viability of cancer cells in a concentration-dependent manner, and it shows a synergistic effect in combination with 5-Fluorouracil (5-FU). **A**. Concentration–response plots for HT-29, HCT-116, Caco-2, A375 treated with increasing concentrations of Lyoph-LGG-SN (up to 5% v/v). MTT assay readout reveals a concentration dependent decrease of cellular viability in the four cancer cell lines. Table on the right summarizes the IC_50_ (% of Lyoph-LGG-SN, v/v) and maximum effect (% viability) calculated per each cell line. **B**. HT-29, HCT-116, Caco-2, A375 concentration response plots. Cells treated with 5-FU (from 5.0 × 10^–7^ M to 5.0 × 10^–4^ M) in combination with different concentrations of Lyoph-LGG-SN (0.3%, 1% and 3% v/v). **C**. HT-29, HCT-116, Caco-2, A375 relative inhibition matrices (left), Highest Single Agent (HSA) synergy matrices (middle), synergy surface with most synergistic area (black square, with corresponding HSA synergy score). N = 3. Values are presented as Mean ± SD

To verify whether LGG-supernatant derived from lyophilized LGG culture medium might show similar effects on cancer cell viability, an MTT assay has been performed, assessing Lyoph-LGG-SN 1:3 serial dilutions starting from 5% v/v. The results shown in Fig. 6A demonstrated that Lyoph-LGG-SN reduced cancer cell viability in a concentration dependent manner. In line with the results obtained with LGG-SN, Caco-2 are the less sensitive cells, with a residual viability of 64% with 5% (v/v) Lyoph-LGG-SN treatment. While, the other three cancer cell lines tested, including melanoma A375 cells, show a residual viability comprised between 37.8% and 25.9%. Correspondingly, the relative IC_50_ values calculated for cancer cell lines range between 2.2% Lyoph-LGG-SN (v/v) for Caco-2 less-sensitive cells, to 0.6% Lyoph-LGG-SN (v/v) and 1.1% Lyoph-LGG-SN (v/v) for HT-29 and A375, respectively; and, finally 1.5% Lyoph-LGG-SN (v/v) for HCT-116 (Fig. 6A, left table).

Combination treatments using 3 different concentrations of Lyoph-LGG-SN in combination with 4 different 5-FU concentrations, showed that Lyoph-LGG-SN has a synergistic effect comparable with LGG-SN, with calculated HSA maximum area mean values of 7.24 for HT-29, 4.65 for HCT-116, 7.36 for Caco-2 and 7.90 for A375 (Fig. 6B, left plots). Overall, these results confirmed that Lyoph-LGG-SN is able to synergize with the anti-cancer drug 5-FU in a way comparable with what observed with LGG-SN.

## Discussion

LGG may be considered a golden bullet in oncology, with yet uncovered dual potential. On one hand, LGG exerts protective effects on human healthy cells, while on the other hand this probiotic is capable of inducing, or fastening, cell death of irreparably damaged cells of the host, such as cancer cells [4]. Regarding colorectal cancer, it has been observed in several preclinical models that LGG administration actively reduced tumor growth, either by contrasting local inflammation or by eliciting tumor shrinkage [26–29]. Also in case of non-intestinal tumors, such as bladder cancer, the administration of live or lyophilized LGG triggered an effective anti-tumor immune response [30–32].

The results hereby reported further suggest that cell-free LGG-SN may selectively reduce cancer cell viability (Fig. 1). This effect has been confirmed also treating cancer cells with cell-free lyoph-LGG-SN (Fig. 6A). Hence, the concentration-dependent reduction of viability observed in cancer cells is specifically triggered by one (or more) molecule or bioproduct secreted by LGG, and, importantly, this effect may be independent from the eukaryotic-specific RPMI-1640 culture media in which the LGG-derived molecules are released/resuspended.

In line with these results, several studies suggested that the LGG-biomolecule(s) with proven anti-cancer activity may be present in LGG cytoplasmic fraction and/or actively released outside. For example, new studies have shown that both HGC-27 gastric cancer and DLD-1 colorectal cancer (CRC) cells were resistant to LGG cell-wall fraction, but sensitive to LGG cytoplasmic one [33, 34]. Also, live LGG inhibited cell growth of Caco-2, HT-29 and SW480 CRC cells [35]. Regarding the downstream effects induced by LGG in cancer cells, short-time incubation with live LGG induced the secretion of zonulin family peptides (potent regulators of intestinal tight junctions) in HT-29 [36]. Also, both live LGG and LGG conditioned medium were capable to inhibit IL-1β-induced IL-8 production and NF-κB signaling pathway activation in Caco-2 [37, 38]. Moreover, cell-free LGG supernatant decreased matrix metallopeptidase 9 (MMP-9) levels and tumor-invasiveness in several CRC cells [39]. Interestingly, it was recently discovered that sterile cell-free supernatant from LGG culture may promote the activity of formyl peptide receptor 1 (FPR1), which is an innate immune sensor of bacteria with anti-inflammatory and anti-angiogenic potential, expressed by both HCT-116 and HT-29 [40]. Moreover, it was proven that LGG-secreted anti-cancer biomolecule(s) may be transported within LGG-derived extracellular vesicles, with the capability to actively reduce the proliferation rate of both HT-29 and SW480 [41]. Also, nanoparticles loaded with LGG cytoplasmic lysate could significantly reduce HT-29 viability and promote apoptosis [42]. In this study, we also demonstrated that both viability and cell death upon LGG-SN treatment were not affected in non-transformed Fibroblasts (Figs. 1 and 2C, D). This result is in line with what previously reported by others, who have shown that LGG had beneficial effects on non-cancerous host’s cells, both in vitro and in vivo. Exosome-like nanoparticles from LGG supernatant might induce *Reg3* and *Nrf2* gene overexpression in mouse intestinal cells, leading to improved barrier function [43]. Analogously, LGG-derived extracellular vesicles attenuated inflammation through inhibition of TLRs/NF-κB/NLRP3 pathway in a murine colitis model [44]. In particular, the LGG-derived protein p40, once secreted, promoted local IgA production, leading to intestinal homeostasis amelioration and anti-inflammatory response [45]. Recent findings demonstrated that healthy intestinal cells may induce LGG to produce and secrete p40 protein, in a positive-feedback loop [46]. Another LGG-secreted protein HM0539 was recently identified as an active factor effectively reducing colitis in rats via activating TLR4/MyD88/NF-кB pathway in enteric cells [47]. Altogether, the observations suggest that the dual effect of LGG might depend on the specific health status of the host’s cell, which may be differentially receptive to either beneficial or toxic bioproducts from LGG.

Pivotally, in contrast with several reported observations, our results evidence that the reduction in viability is not associated with cell death in cancer cells upon LGG-SN treatment (Fig. 2). In particular, neither apoptosis (early or late) nor necrosis is activated in cancer cells upon LGG-SN treatment (Additional file 1: Figs. S1 and S2). This dissimilarity may be due to the diverse LGG-SN enrichment protocol hereby used when compared to others. Our approach allows to get rid of potentially detrimental factors (or possibly low pH) of MRS medium. We obtained LGG-conditioned RPMI-1640 complete medium, which is optimal for eukaryotic cell growth. In our hands, as shown in Additional file 1: Fig. S5, MRS medium alone is cytotoxic, even when used at lowest percentages, therefore it is impossible to use LGG-conditioned MRS complete medium on eukaryotic cells. It was reported by others that treatments with either LGG supernatant produced in MRS medium or live LGG were associated with cell death induction in tumors. For example, culture supernatant of *L. rhamnosus* produced in MRS induced a cell cycle arrest in HT-29 cells accompanied by increased pro-apoptotic gene expression [48]. Also, cervical cancer cells ME-180, when incubated with live *L. rhamnosus* arrest their cell cycle with concurrent nuclear accumulation of p21 protein [49]. Live LGG induced a cell cycle arrest in G0/G1 phase associated with increased apoptosis in both HT-29 and Caco-2 cells [35]. Contrariwise, *L. plantarum* conditioned medium obtained with a protocol similar to ours (based on the utilization of bacteria-conditioned cell-free eukaryotic cell culture media) induced a reduction of viability in HT-29 not associated with apoptosis, but with a G2/M cell cycle arrest [50]. Besides, *L.* *pentosus* and *L.* *plantarum* RPMI-based conditioned cell-free media induced a G0/G1 cell cycle arrest associated with reduction of viability, but not cellular death in HT-29 and Caco-2 cells [51].

Our results demonstrated that the reduction in viability observed in cancer cells following LGG-SN treatment, is associated with a significant increase in the percentage of cancer cells blocked in G2/M phase of the cell cycle and a decrease in the number of cells in G1 phase, without any significant change in percentage of dead cells detected in Sub-G1 area. The effect observed with LGG-SN in cancer cells was similar to what obtained with anti-mitotic drugs such as VIN, here used as positive control (Fig. 3A and Additional file 1: Fig. S4). In fact, mitotic cell cycle arrest may be not necessarily associated with concurrent cell death induction, but it can trigger a cytostatic effect such as cell cycle arrest/senescence [52]. The observed cytostatic effect induced by LGG-SN might explain the detected increased expression of Cyclin A, Cyclin B and Cyclin D genes compared with CTRL levels (Fig. 3B) [53–55]. However, the biological significance of such expression changes, especially in case of small increases (i.e., Cyclin B gene expression in LGG-SN treated HT-29 compared with CTRL) will deserve further validation in the future, with functional experiments.

Finally, this study aimed to shed light on the adjuvant potential of cell-free LGG-SN in anti-cancer therapy. We employed both 5-FU and IRN, both widely used in clinics and, importantly, both associated with possible overcoming resistance in subgroups of patients. These data suggest a strong need for finding novel effective tumor-targeting approaches [56, 57]. The results hereby obtained demonstrate that LGG-SN significantly increases the anti-proliferative effect of two main anti-cancer drugs, 5-FU and IRN, largely employed in clinics (Figs. 4, 5) [58, 59]. In particular, both LGG-SN and lyoph-LGG-SN effectively sensitize cancer cells to 5-FU anti-cancer drugs (Figs. 4A and 6B). Additionally, LGG-SN sensitizes tumor cells to IRN (Fig. 5A). The HSA scoring assumes that the potential combination effect equals to the higher individual drug effect, thereby supporting that a synergistic drug combination should produce additional benefits on top of what its single drug compounds can achieve alone [23]. According to that, for each combined treatment tested (LGG-SN in combination either with 5-FU or IRN), and for every cancer cell line utilized, it was possible to calculate a main synergistic area with positive HSA values within the interaction landscapes (Figs. 4B and 5B). Interestingly, when combination assays were performed by using lyoph-LGG-SN in combination with 5-FU the synergy results were equally confirmed, with comparable calculated HSA maximum and mean values (Fig. 6B and D). This latter result confirms that when lyophilized, LGG-secreted molecule(s) maintain the same anti-proliferative effect than when released in RPMI-1640 complete medium and they are capable to sensitize cancer cells to 5-FU cytotoxic agent (Fig. 6).

According to our results, a recent study suggested that both live and heat-killed LGG may be able to sensitize 5-FU treated Caco-2 CRC cells by inducing the expression of TNF-α, MCP-1, and IL-1 genes [60]. This effect appears to be selective for cancer cells, as non-transformed IEC-6 rat intestinal epithelial cells treated with 5-FU in combination with LGG supernatant showed on the contrary a reduction in pro-apoptotic caspase expression [61].

In conclusion, current research is trying to understand what are the LGG-bioproducts either responsible for beneficial or detrimental effects exerted on host’s cells and tissues. What makes the difference in the nature of the response observed in human LGG-targeted cells? Available data suggest that the health status of the human target cells might make a crucial difference, as non-transformed cells seem to be protected by LGG, while cancer cells seem to be negatively affected by LGG. Several LGG-derived molecules with anti-apoptotic activity toward non-cancer cells have been identified, including p40, p75, HM0539 and bacteriocins [47, 62, 63]. Also, LGG can secrete high concentrations of short-chain fatty acids (acetate, propionate, butyrate) which are beneficial for the maintenance of the host intestinal homeostasis [64]. Regarding the LGG-anti-cancer activity, apart from LGG-derived lipoteichoic acid, no other molecule with anti-proliferative effect has been identified yet [65]. Importantly, the strategy used to produce LGG (or its supernatant) may affect the profile of biomolecules and metabolites synthetized by the bacteria. For that reason, it is very important to consider, for example, the specificity of the interaction occurring between LGG and the rest of the GM, which might change the pH and other parameters within the intestinal milieu. Also, from the industrial point of view, it may be pivotal to select the best growth media in correlation with the specific biomolecules whose quantity/quality needs to be maximized, depending on the downstream application [66–68].

## Conclusion

Our results suggest that LGG-SN may contain one or more bioactive molecules with anti-cancer activity which sensitize cancer cells to chemotherapeutic drugs. Thus, LGG could be proposed as an ideal candidate for new appealing integrated approaches in oncology, which might help to overcome resistance or relapse issues and, overall, to ameliorate the outcomes of cancer patients. Future studies are strongly needed to assess the specific nature of LGG-derived active biomolecule(s), and how such mediators might be differentially sensed by human cells, based on their specific health status.

## Supplementary Information

**Additional file 1: Figure S1.** Cancer cells treated with LGG-SN do not undergo apoptosis: immunoblot analysis. Immunoblot and densitometry of A375, Caco-2, HCT-116, HT-29 treated with 0% v/v LGG-SN (CTRL), 90% v/v LGG- SN, 0.5 µg/ml Puromycin (positive control treatment). Signal detected and measured via densitometry analysis for cleaved Caspase 3 (c-Casp-3; 17–19 KDa), full-length PARP (t-PARP; 116 KDa) and cleaved PARP (c-PARP; 89 KDa), β-Actin (normalization control, 42 KDa). Values are presented as Mean ± SD. **Figure S2.** Cancer cells treated with LGG-SN do not undergo apoptosis or necrosis: flow cytometry analysis. A. Dot-plot cytograms of Annexin-V (ch02) versus Propidium Iodide (ch05) fluorescence intensity in A375, Caco-2, HCT-116, HT-29 tumor cells treated with 0% v/v LGG-SN (CTRL), 90% v/v LGG-SN (LGG-SN), 5 × 10–7 M Vincristine (VIN), and in HT-29 treated with 0.5 µg/ml Puromycin (PURO). B. Cell death analysis bar plots: percentage of cells live (yellow), necrotic (orange), apoptotic (red) and early apoptotic (pink). C. Representative Flow Sight images of cells live, early apoptotic, apoptotic, necrotic. BF, Bright field. N = 3. Values are presented as Mean ± SD. Statistical significance was analyzed using two-way ANOVA with Tukey's multiple comparisons test. * p < 0.05; *** p < 0.001; **** p < 0.0001; no asterisk = not significant. **Figure S3.** Cancer cells treated with LGG-SN show a decrease in cell number. HT-29, HCT-116, Caco- 2, A375 and Fibroblasts were treated either with 0% v/v LGG-SN (CTRL, grey bars) or 90% v/v LGG-SN (LGG-SN, pink bars). Doubling times were calculated 48 h after treatment. N = 4. Values are presented as Mean ± SD. Statistical significance was analyzed using two-way ANOVA with Šídák's multiple comparisons test. *** p < 0.001; **** p < 0.0001; n.s. = not significant. **Figure S4.** Cell cycle analyses reveal G2/M block upon LGG-SN treatment in cancer cells. HT-29, HCT-116, Caco-2, A375 treated with 0% v/v LGG-SN (CTRL), 90% v/v LGG-SN and 5 × 10–7 M Vincristine (VIN). Cell cycle flow cytometry histogram plots with counted events expressed as normalized frequency function of Propidium Iodide fluorescence intensity (Ch05). Tables reported summarize per each plot the absolute frequency (Count) and relative percentage frequency (% Gated) of events (cells) counted in different phases of cell cycle (corresponding to different DNA content and different fluorescence intensities). R1, region gating cells in G0/G1 phase; R2, region gating cells in S phase; R3, region gating cells in G2/M phase; R4, region gating cells in Sub-G0 phase. **Figure S5.** Differential viability effect of MRS and RPMI conditioned media. Comparison of viability in cancer cells when treated with different concentrations of LGG supernatant in RPMI-1640 (LGG-SN RPMI), MRS (LGG-SN MRS) and MRS mock control (MRS only not incubated with LGG). N = 3. Values are presented as Mean ± SD.

## Data Availability

All data are available in the manuscript or upon request to the authors. Supplementary and raw data were also deposited on Zenodo (https://doi.org/10.5281/zenodo.7697382).

## Abbreviations

GM: Gut microbiota
LGG: *Lactobacillus rhamnosus* GG
LGG-SN: Cell-free LGG supernatant
Lyoph-LGG-SN: LGG lyophilized cell-free and sterile supernatant
