# Supplementary material for: Lactobacillus rhamnosus GG cell-free supernatant as a novel anti-cancer adjuvant
Source: J Transl Med. 2023 Mar 14;21:195. doi: 10.1186/s12967-023-04036-3 (PMC10015962; doi:10.1186/s12967-023-04036-3)

Additional file

Additional **Fig. S1. Cancer cells treated with LGG-SN do not undergo apoptosis: immunoblot analysis.** Immunoblot and densitometry of A375, Caco-2, HCT-116, HT-29 treated with 0% v/v LGG-SN (CTRL), 90% v/v LGG- SN, 0.5 µg/ml Puromycin (positive control treatment). Signal detected and measured via densitometry analysis for cleaved Caspase 3 (c-Casp-3; 17-19 KDa), full-length PARP (t-PARP; 116 KDa) and cleaved PARP (c-PARP; 89 KDa), β-Actin (normalization control, 42 KDa). Values are presented as Mean  ±  SD.


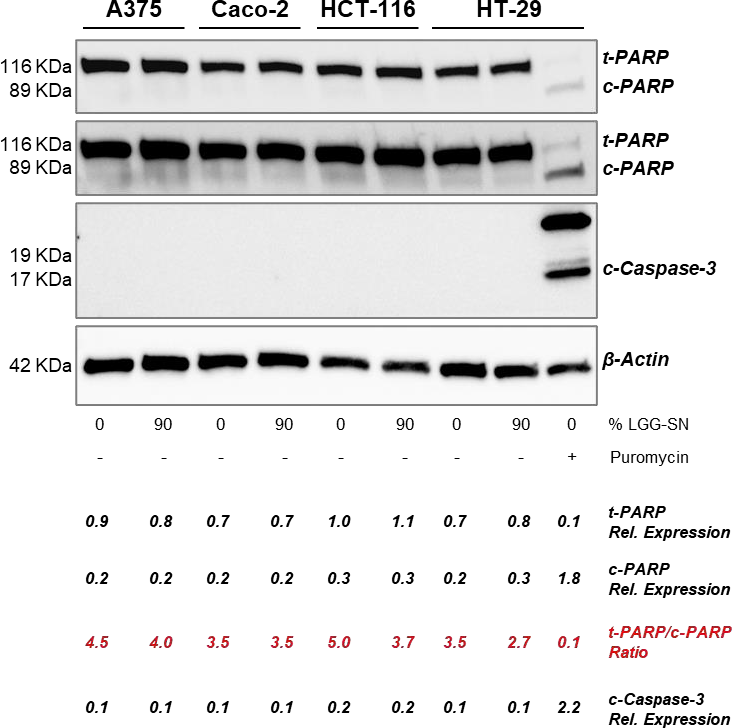


Additional **Fig. S2. Cancer cells treated with LGG-SN do not undergo apoptosis or necrosis: flow cytometry analysis. A.** Dot-plot cytograms of Annexin-V (ch02) versus Propidium Iodide (ch05) fluorescence intensity in A375, Caco-2, HCT-116, HT-29 tumor cells treated with 0% v/v LGG-SN (CTRL), 90% v/v LGG-SN (LGG-SN), 5 x 10^-7^ M Vincristine (VIN), and in HT-29 treated with 0.5 µg/ml Puromycin (PURO). **B.** Cell death analysis bar plots: percentage of cells live (yellow), necrotic (orange), apoptotic (red) and early apoptotic (pink). **C.** Representative Flow Sight images of cells live, early apoptotic, apoptotic, necrotic. BF, Bright field. N = 3. Values are presented as Mean  ±  SD. Statistical significance was analyzed using two-way ANOVA with Tukey's multiple comparisons test. * p < 0.05; *** p < 0.001;

**** p < 0.0001; no asterisk = not significant.


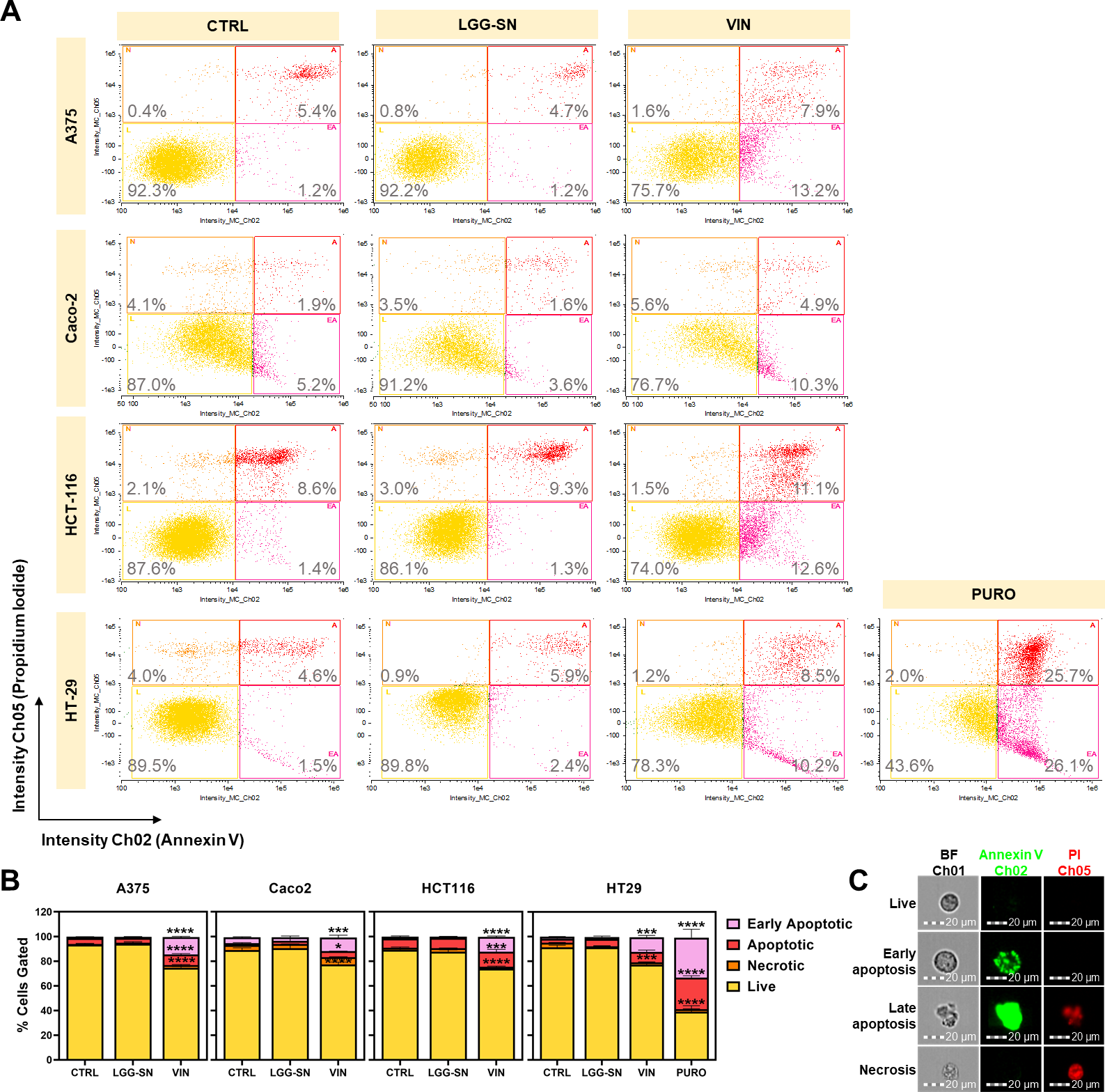


Additional **Fig. S3. Cancer cells treated with LGG-SN show a decrease in cell number.** HT-29, HCT-116, Caco- 2, A375 and Fibroblasts were treated either with 0% v/v LGG-SN (CTRL, grey bars) or 90% v/v LGG-SN (LGG-SN, pink bars). Doubling times were calculated 48 hours after treatment. N = 4. Values are presented as Mean  ±  SD. Statistical significance was analyzed using two-way ANOVA with Šídák's multiple comparisons test. *** p < 0.001; **** p < 0.0001; n.s. = not significant.


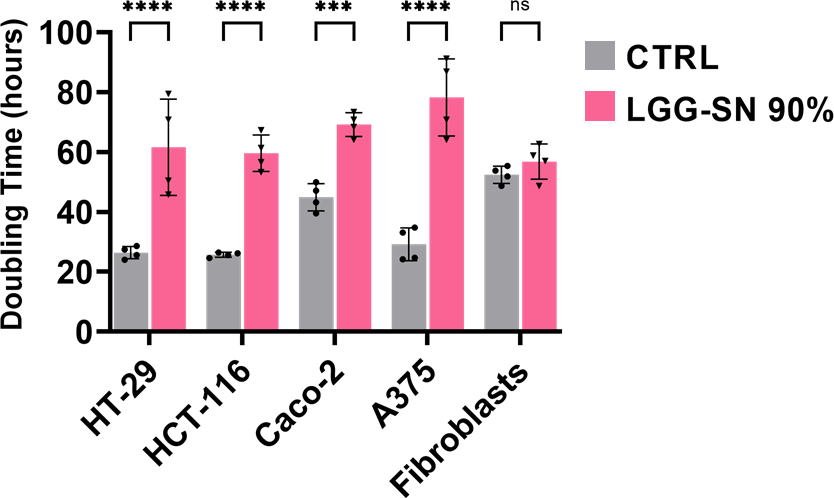


Additional **Fig. S4. Cell cycle analyses reveal G2/M block upon LGG-SN treatment in cancer cells.** HT-29, HCT-116, Caco-2, A375 treated with 0% v/v LGG-SN (CTRL), 90% v/v LGG-SN and 5 x 10^-7^ M Vincristine (VIN). Cell cycle flow cytometry histogram plots with counted events expressed as normalized frequency function of Propidium Iodide fluorescence intensity (Ch05). Tables reported summarize per each plot the absolute frequency (Count) and relative percentage frequency (% Gated) of events (cells) counted in different phases of cell cycle (corresponding to different DNA content and different fluorescence intensities). R1, region gating cells in G0/G1 phase; R2, region gating cells in S phase; R3, region gating cells in G2/M phase; R4, region gating cells in Sub-G0 phase.


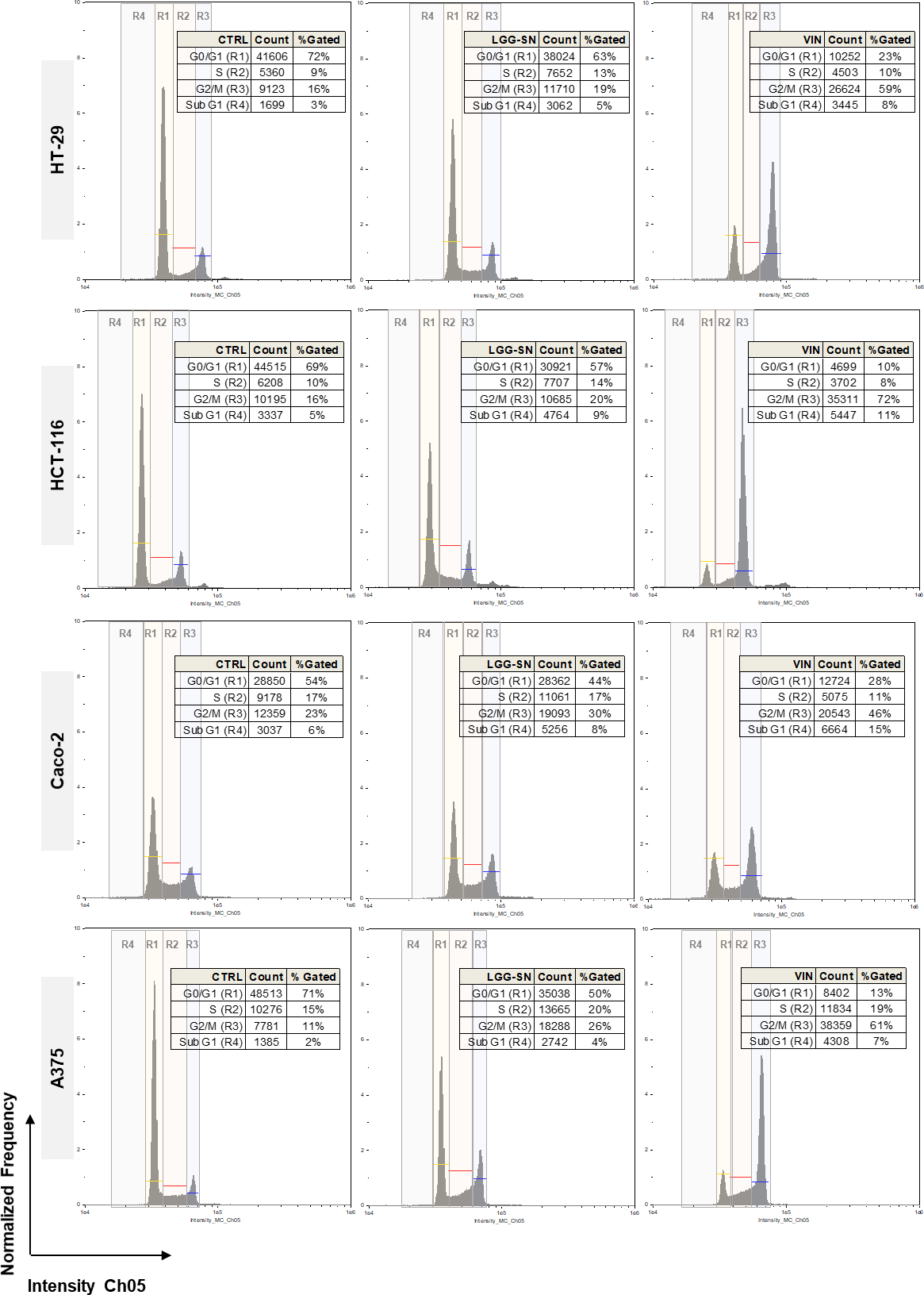


Additional **Fig. S5. Differential viability effect of MRS and RPMI conditioned media.** Comparison of viability in cancer cells when treated with different concentrations of LGG supernatant in RPMI-1640 (LGG-SN RPMI), MRS (LGG-SN MRS) and MRS mock control (MRS only not incubated with LGG). N = 3. Values are presented as Mean  ±

 SD.


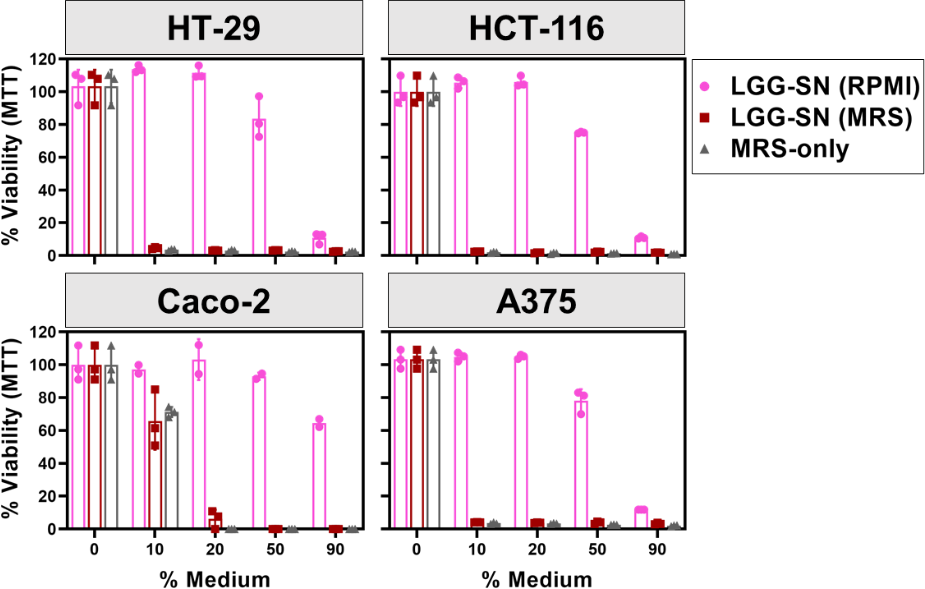

Supplement: Supplementary file 1 — Additional file 1: Figure S1. Cancer cells treated with LGG-SN do not undergo apoptosis: immunoblot analysis. Immunoblot and densitometry of A375, Caco-2, HCT-116, HT-29 treated with 0% v/v LGG-SN (CTRL), 90% v/v LGG- SN, 0.5 µg/ml Puromycin (positive control treatment). Signal detected and measured via densitometry analysis for cleaved Caspase 3 (c-Casp-3; 17–19 KDa), full-length PARP (t-PARP; 116 KDa) and cleaved PARP (c-PARP; 89 KDa), β-Actin (normalization control, 42 KDa). Values are presented as Mean ± SD. Figure S2. Cancer cells treated with LGG-SN do not undergo apoptosis or necrosis: flow cytometry analysis. A. Dot-plot cytograms of Annexin-V (ch02) versus Propidium Iodide (ch05) fluorescence intensity in A375, Caco-2, HCT-116, HT-29 tumor cells treated with 0% v/v LGG-SN (CTRL), 90% v/v LGG-SN (LGG-SN), 5 × 10^–7^ M Vincristine (VIN), and in HT-29 treated with 0.5 µg/ml Puromycin (PURO). B. Cell death analysis bar plots: percentage of cells live (yellow), necrotic (orange), apoptotic (red) and early apoptotic (pink). C. Representative Flow Sight images of cells live, early apoptotic, apoptotic, necrotic. BF, Bright field. N = 3. Values are presented as Mean ± SD. Statistical significance was analyzed using two-way ANOVA with Tukey's multiple comparisons test. * p < 0.05; *** p < 0.001; **** p < 0.0001; no asterisk = not significant. Figure S3. Cancer cells treated with LGG-SN show a decrease in cell number. HT-29, HCT-116, Caco- 2, A375 and Fibroblasts were treated either with 0% v/v LGG-SN (CTRL, grey bars) or 90% v/v LGG-SN (LGG-SN, pink bars). Doubling times were calculated 48 h after treatment. N = 4. Values are presented as Mean ± SD. Statistical significance was analyzed using two-way ANOVA with Šídák's multiple comparisons test. *** p < 0.001; **** p < 0.0001; n.s. = not significant. Figure S4. Cell cycle analyses reveal G2/M block upon LGG-SN treatment in cancer cells. HT-29, HCT-116, Caco-2, A375 treated with 0% v/v LGG-SN (CTRL), 90% v/v LGG-SN and [file 12967_2023_4036_MOESM1_ESM.docx]
